# Sub-Gigahertz Path Loss Measurement Campaign in Marine Environment: A Case Study

**DOI:** 10.3390/s24082582

**Published:** 2024-04-18

**Authors:** Marco De Piante, Michele Midrio, Roberto Rinaldo, Ivan Scagnetto, Ruben Specogna, Francesco Trevisan

**Affiliations:** 1 Polytechnic Department of Engineering and Architecture, University of Udine, via delle Scienze 206, I-33100 Udine, Italy; marco.depiante@uniud.it (M.D.P.); michele.midrio@uniud.it (M.M.); roberto.rinaldo@uniud.it (R.R.); francesco.trevisan@uniud.it (F.T.); 2 Department of Mathematics, Informatics and Physics, University of Udine, via delle Scienze 206, I-33100 Udine, Italy; ivan.scagnetto@uniud.it

**Keywords:** data communication, marine environment, path loss, sub-gigahertz bands

## Abstract

This paper focuses on the characterization of radio propagation, and data communication in a marine environment. More specifically, we consider signal propagation when three different sub-gigahertz industrial, scientific, and medical (ISM) bands, i.e., 169 MHz, 434 MHz, and 868 MHz, are used. The main focus of the paper is to evaluate the path loss (PL), i.e., the power loss that a propagation radio wave would experience when communication occurs between a sail boat and a buoy. We describe the measurement results obtained performing three different radio power measurement campaigns, at the three different aforementioned ISM sub-gigahertz bands. We also want to correlate the radio propagation quality with the weather conditions present in the measurement areas. The obtained results show that higher distances are achieved by transmitting at lower frequencies, i.e., 169 MHz, and, on average, the propagation is directly dependent from the dew point index.

## 1. Introduction

Nowadays, automation and internet of things (IoT) are quickly spreading across maritime applications [1]. Vessels, buoys, and other kinds of objects, capable of moving or simply floating on the surface of the sea, are filled up with sensors [2]. The latter produce a wealth of data that must be transmitted to suitable receivers, which process such information. Common application scenarios are environmental monitoring campaigns, where buoys are equipped with probes sensing the quality of water [3,4], or sailing boats regattas (even at amateur level), where the race management is, at least partially, automated (for instance, in tracking vessels positions and in assigning points to participants). Such applications usually require one to transmit data across several miles, often without the availability of Internet connections. A suitable solution for transferring a small number of data is the deployment of satellite connections [5]. A cheaper but reliable and robust alternative is the deployment of radio devices that exploit the sub-gigahertz bands [6]. This paper is focused on data communication and transmission in a marine environment. More specifically, we want to carry out a path loss (PL) measurement campaign [7] when communication occurs between a sail boat and a buoy. An example of a similar measurement approach is reported in [8], where the authors performed a comprehensive analysis of the propagation that occurs in the marine environment for frequencies in the 5 GHz band. The scope of this paper is to present the measurement setup that allowed us to determine the sub-gigahertz propagation PL: it consists of the design and implementation of antennas and radio frequency (RF) transceivers, as well as the adopted storage and data logging choices. The measurement campaign has been carried out in three different sub-gigahertz industrial, scientific, and medical (ISM) bands, i.e., 169 MHz, 434 MHz, and 868 MHz, which, to the author’s knowledge, are not fully investigated in the literature for this application. As mentioned above, in these three different transmission frequencies we focus our attention on the PL between the two antennas: this allowed us to outline the pros and cons that a communication system sees in such an environment and differentiate the performance when different frequencies are chosen. The PL and its implications are of fundamental importance for understanding the propagation conditions in the marine environment, and this leads to the generation of rules-of-thumb when designing new radio devices that will operate in such scenarios. In this paper, we want to point out another key point that is not fully investigated in the literature. Due to the fact that, by fixing the environmental scenarios (i.e., sail boat–bouy distances, measurement locations), the measurements exhibit a certain performance variability in terms of PL, we corroborate the measurements’ results, introducing an investigation into the weather conditions of the measurement scenarios, correlating the propagation conditions with the atmospheric parameters. The results are presented in an empirical form, which clearly indicates communication performance dependence with respect to the weather conditions. The paper is organised as follows. In Section 2, we show the adopted hardware, in terms of either digital boards and RF equipment. In Section 3, we analytically describe the propagation channel we considered, retrieving rules-of-thumb for the geometry of the measurement system. In Section 4, we present the measured data in terms of both path loss exponent and fluctuations around the average values, with a digression on the statistical description of the retrieved channel model. In Section 5, we correlate the path loss exponent with measured weather parameters, in order to achieve propagation information observing the atmospheric conditions. Then, the conclusion follows.

## 2. Measurement Test-Bed

In this section, we describe the architecture of the two communicating nodes, which allowed us to characterize the sub-gigahertz propagation signals in the marine environment. We start by describing the antennas we used, and then we describe the RF and baseband devices, as well as the storage equipment we deployed for data acquisition.

### 2.1. Antenna Design

**Figure 1:**
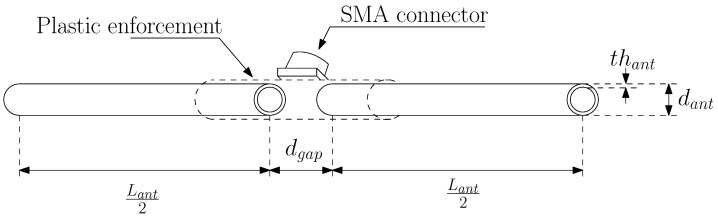
Geometry and physical quantities of the deployed dipoles (also depicted).

**Figure 2:**
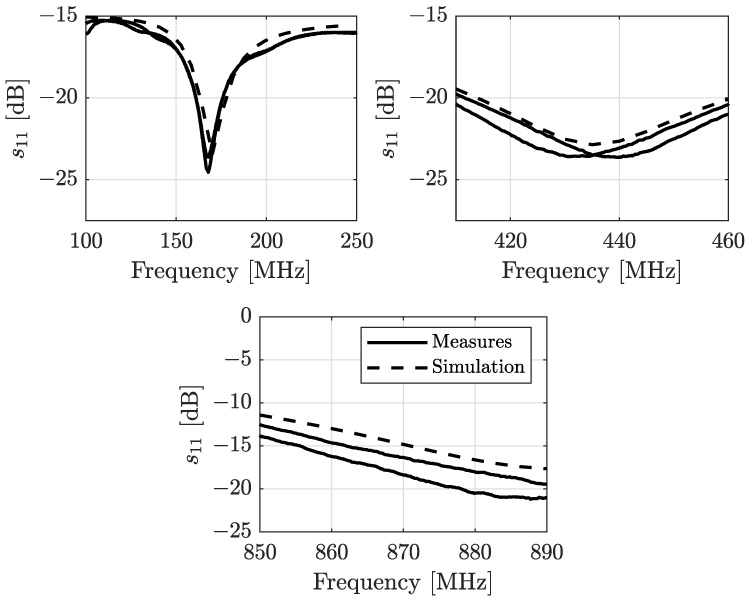
Measured and simulated return loss in dB of the three different pairs of deployed dipole antennas.

In this subsection, we detail the characteristics of the three different antennas we deployed for the measurement campaign. We decided to use a couple of dipole antennas for each band, tuned to be matched with a 50 Ω transmission line, i.e., the coaxial cable that connects the antenna with the transceiver. This decision was been made since the three different ISM bands we took into consideration have their allowable transmission bandwidth restricted to 12.5 kHz at 169 MHz and to a maximum of 100 kHz for the remaining 434 MHz and 868 MHz bands. A simple dipole antenna exhibits a narrow resonance band [9] (i.e., the frequencies in which the antenna efficiently delivers the power to the electromagnetic field in the propagation medium), which is sufficient for the purpose of our transmission system. The geometry and the physical dimensions are drawn in Figure 1. Basically, the dipole is composed of two tubular aluminium arms of length $L_{ant}/2$ and diameter $d_{ant}$ inserted into a thin plastic enforcement. The extremities are separated by a gap distance $d_{gap}$. The two arms are connected to a SubMiniature version A (SMA) female connector: one to the hot pin and one to the ground pin. The diameter $d_{ant}$ is the same for the three antennas and is equal to 6 mm with a thickness $th_{ant}$ of 0.7 mm. The $d_{gap}$ distance is 5 mm. The $L_{ant}/2$ is equal to 411 mm, 154 mm, and 71 mm for the 169 MHz, 434 MHz, and 868 MHz dipoles, respectively. With the use of a vector network analyzer (VNA) to collect the measurements, in Figure 2 we show both the measured and simulated return loss (RL) [10] or, equivalently, the $s_{11}$ magnitude scattering parameter, of the three different designed dipoles. The values are expressed in dB scale; thus,

(1) $$\mathrm{RL}=20\log_{10}\left(\right|s_{11}\left|\right),$$

where we can assume that the scattering parameter $s_{11}$ is related to the reflection coefficient Γ, which is defined as

(2) $$\Gamma =\frac{Z_{ant}-Z_{0}}{Z_{ant}+Z_{0}}.$$

In the mentioned expression, $Z_{ant}$ is the antenna impedance exhibited at the considered frequency, and $Z_{0}$ is the reference impedance or, equivalently, the characteristic impedance of the transmission line used to connect the antenna to the VNA, as well as the VNA internal port impedances. It is easy to understand that the reflection coefficient should be close to zero (or, equivalently, RL →−∞), so all the power supplied by the transmitter is radiated by the antenna. The simulation has been carried out in the Ansys HFSS simulator [11].

The RL measurements show good adherence with the results obtained through the simulations, in particular for the return loss related to the 169 MHz dipole. The other two dipoles have better RL than the simulations indicate, and all of the three deployed dipoles exhibit a RL lower than −15 dB, which corresponds to a reflected power percentage less than 3.2%.

### 2.2. Antenna System

**Figure 3:**
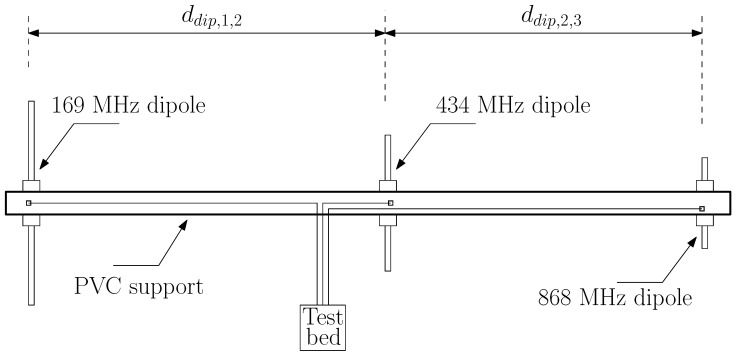
Dipole system geometry.

**Figure 4:**
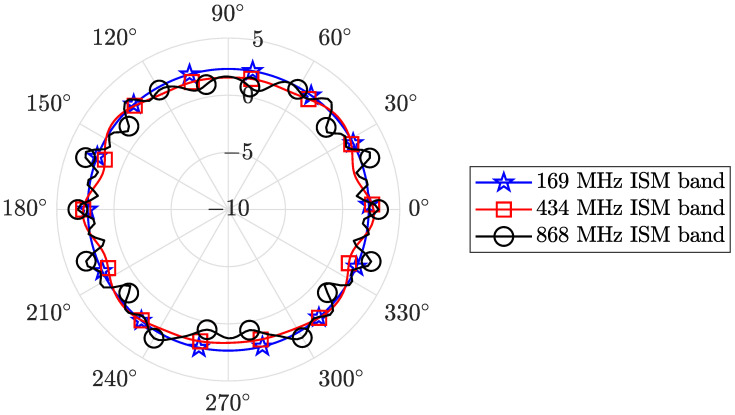
Gain pattern at the three different ISM bands for the dipole system of Figure 3.

The three different dipoles, one for each different considered ISM band, are collected together in an array and fixed on a plastic support as shown in Figure 3. The plastic support length is 2 m, and its diameter is 30 mm, with a thickness of 1.5 mm. In order to minimize parasitic effects among the dipoles in the system (i.e., an undesired gain pattern for each dipole), as well as to maintain a compact geometry for the system, the distance between the 169 MHz dipole and the 434 MHz one is $d_{dip,1,2}=1070$ mm, while the distance between the 434 MHz dipole and the 868 MHz one is $d_{dip,2,3}=870$ mm. Since we do not have the possibility to measure the actual gain pattern for the system, for example, in an anechoical chamber suitable for the 169 MHz frequencies, Figure 4 reports the results of the simulated gain pattern, for the three considered bands. As is noticeable, the depicted gains show a slight oscillation around the mean value. Table 1 shows the average gain $G_{avg}$ of the system, along with the minimum gain $G_{min}$ and maximum gain $G_{max}$ values for each ISM band obtained via simulations.

### 2.3. Rf and Baseband Test Bed

**Figure 5:**
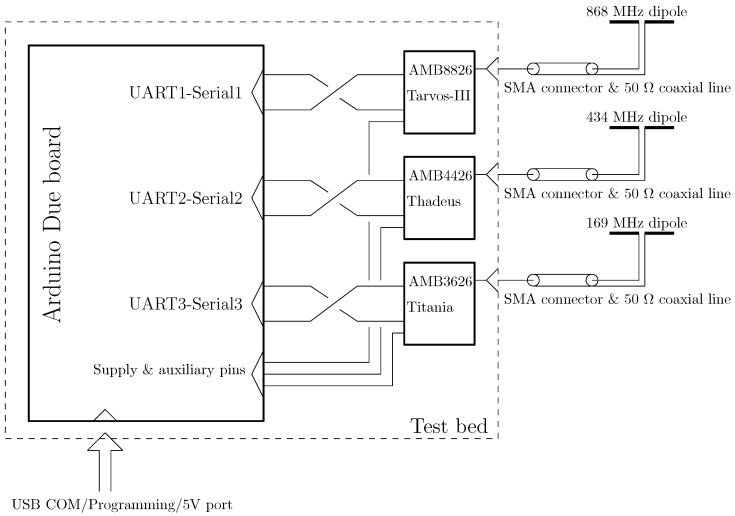
Test bed deployed in the measurement campaign. The general digital architecture is displayed, as well as the connections among the antennas and the radio modules. The supply voltage is set to be 5 V, obtained by a lithium battery and a voltage regulator.

For the purpose of this study, we decided to use three different radio modules (RMs) as transceiver nodes, one for each considered ISM band. In particular, we deployed three Wurth Elektronik RF sub-modules [12] belonging to the same RM family. The 169 MHz ISM band is covered with the AMB3626 Titania module; the 434 MHz ISM band is covered with the AMB4426 Thadeus module; and, finally, the 868 MHz ISM band is covered with the AMB8826 Tarvos-III module. Each of them has a universal asynchronous receiver-transmitter (UART) port to be used either for programming and for data transmitting/receiving. For the baseband data management, we equipped the RF front end with an Arduino Due-based module that can handle these three RMs simultaneously. In fact, the Arduino Due board is capable of both supplying the RMs with 3.3 V and interfacing with the three native 3.3 Volt-based UARTs.Further, the Arduino Due board has several pins that become useful for the RM programming interface. Figure 5 shows the schematic block diagram of the designed test bed. Note that the same test bed is deployed on both the sail boat and the buoy. Each of the RMs can receive a set of commands through the UART port that can be used to set the RF parameters, i.e., transmission power, transmitter/receiver frequency, and so on. The RMs have a common UART instruction set, and in particular, when an RM receives a data packet, the UART buffer will be filled with the received data plus the received signal strength intensity (RSSI) that the module has detected. The RSSI will be fundamental for our measurement campaign. In order to obtain a robust communication protocol, we implemented a bidirectional communication for the RSSI measurements. In particular, considering the test bed on the sail boat, we programmed the Arduino board to send a request packet for each frequency every second. The remote buoy test bed receives the packets and sends back a known data packet, which contains the RSSI information. Then, the test bed on the sail boat will receive the data packet and will communicate the RSSI for each ISM band through the USB/COM port. In order to monitor the losses in between the RMs and the antenna system, we also measured the RM-antenna coaxial cable losses in terms of scattering parameters.

In particular, we adopted the scattering parameter $s_{2}1$ expressed in decibels. As reported in [10], the insertion loss (IL) or, equivalently, the $s_{21}$ scattering parameter is related to the reflection coefficient (2): physically, the IL can be assumed to represent the transmission coefficient τ of a device. For passive components like the cable we are testing, the IL represents the attenuation of a device, and it can be expressed in decibels as

(3) $$\tau =1+\Gamma \Rightarrow \mathrm{IL}=s_{21}=20\log_{10}\left(|\tau |\right)\;\left[\mathrm{dB}\right].$$

**Figure 6:**
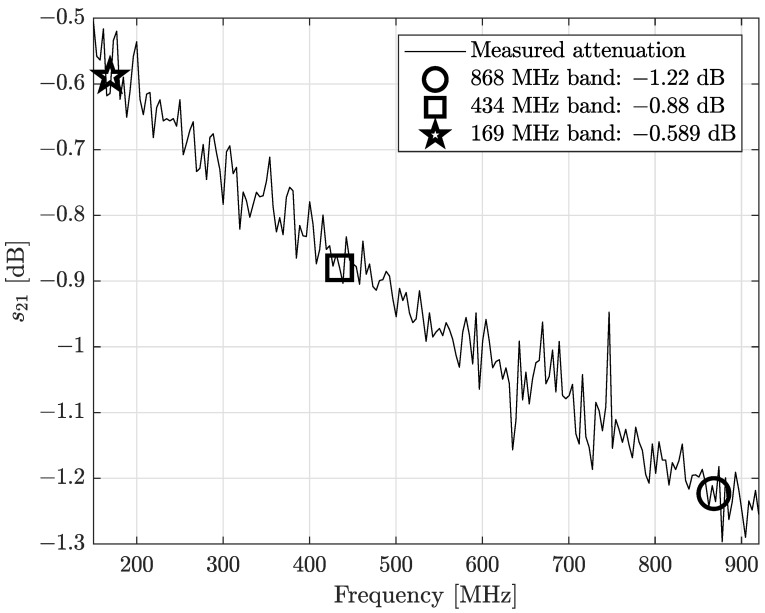
Antenna-RM connection cable losses, measured in terms of insertion loss (or, equivalently, transmission coefficient |τ|) and expressed in decibels.

The results are reported in Figure 6, where we marked the cable gain at the three considered ISM bands. The IL of a 2 m long cable exhibits slight power losses, and, as expected, the losses increase when the frequency increases.

### 2.4. Rf Power and Transmission Frequencies

**Figure 7:**
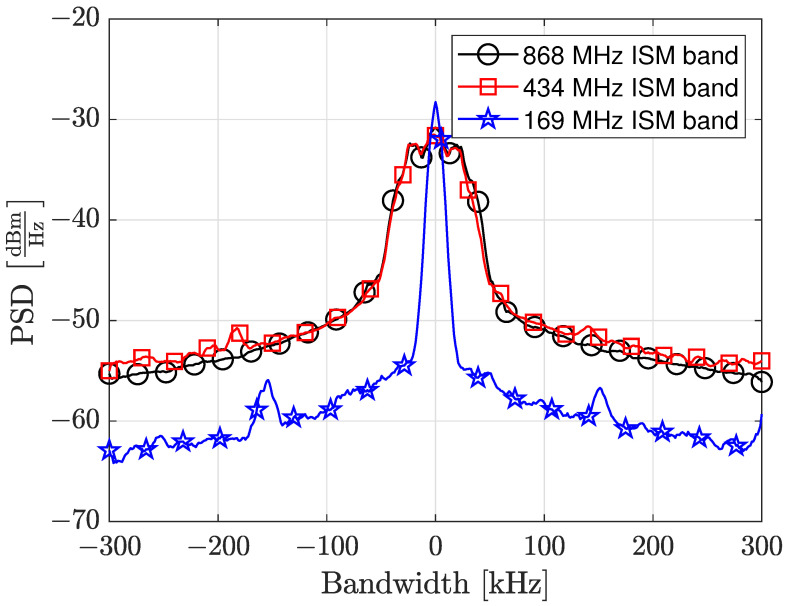
Transmitted PSDs at the three considered ISM bands. Notice that the integral over the considered bandwidth remains almost constant as expressed in the Table 2.

For the measurement campaign, we set the total transmitted RF power to be the same for the three ISM bands. This means that the integral of the power spectral density (PSD) of the transmitted signal at the output RF port of each RM should be the same. Using the PSD measurement guidelines in ETSI EN 300 220-1 [13], we have connected each RM at the input port of a calibrated spectrum analyzer. The PSD measure has been carried out using the power meter application of the spectrum analyzer, which permits one to automatically determine the band in which the 99% of the measured power resides. The maximum allowable RF power (the integral of the PSD) for the 868 MHz ISM band is set to 14 dBm, which is the strictest level for the three considered ISM bands. Consequently, this will represent the power that the other two RMs should deliver to the antenna, in order to maintain the total RF power to be the same for all the tested bands. The three different PSDs we measured at the output RF port of each RM are shown in Figure 7.

Table 2 shows the measured occupied bandwidth (OBW) and the measured channel power (ChP) with the declared parameters, in accordance with the recommendations indicated in ETSI EN 300 220-1 V3.1.1 clause 5.6.3.

### 2.5. Remarks about the Telemetry and Communication System

As far as the software for the telemetry and communication system is concerned, we reused an ad hoc version of the Oceanus infrastructure described in [14], in order to limit the power consumption (especially on the buoy) while retaining the capability to easily change settings on-the-fly during the tests. Hence, we installed a Raspberry Pi 3 (model B+) device both on the boat and on the buoy, connected through a USB port to the Arduino-Due boards used to interface the radio modules previously described. The power supply was a 24 Ah power bank that, according to our experience, allows the system to stay up for 9 h. The Raspberry Pi allows us to:

1. Harvest raw data from sensors (connected through USB ports, and the i2c bus), e.g., GPS coordinates, angle w.r.t. magnetic north, wind speed, and angle;
2. Compute meaningful and useful information from raw data;
3. Provide a WiFi local area network (LAN);
4. Publish the computed information through a socket service and a web application in the LAN;
5. Send or receive coordinates and wind data via sub-GHz radios.

Thus, buoys equipped with this hardware (enclosed in a water proof plastic encasing) can communicate their positions and the related wind speed and angle. Such information will be gathered by another Raspberry Pi node acting as a receiver onboard of the boat.

Moreover, human operators, moving onboard their rubber boats, can easily monitor and control the settings of the buoy upon entering the LAN coverage range provided by the Raspberry Pi device using their smartphones, tablets, etc. (via the above-mentioned web application).

## 3. Radio Channel Analytical Description

**Figure 8:**
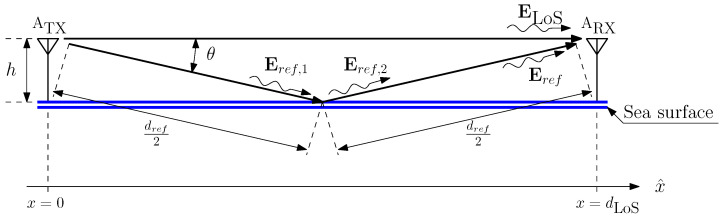
Geometry for the radio channel analysis.

In this section, we analyze some characteristics of the radio channel in a marine environment. Figure 8 summarizes the situation that the two ISM nodes see. Basically, the transmitter antenna $A_{\mathrm{TX}}$ delivers power to the electromagnetic field. In accordance with the antenna gain pattern, a portion of the transmitted power is sent towards the sea surface, causing a reflection. This brings one to have a replica of the transmitted signal that reaches the receiver antenna $A_{\mathrm{RX}}$, causing interference at the receiver module. These kinds of channels are referred to as two-ray channels, and their behavior has been fully investigated in [7], where a typical channel response is plotted. It is easy to verify how the power decay may exhibit strong valleys when the RF nodes are separated by short distances. On the other hand, if the two RF nodes are separated by great distances, the channel gain exhibits a smooth magnitude response, which monotonically decreases as the distance increases. In the latter case, the channel gain linearly decreases if the antennas’ separation distance is considered in logarithmic units. For the purpose of this paper, we are interested in retrieving the power decay slope of the channel gain. To do so, we need to set a threshold distance beyond which the channel response does not exhibit power drops.

Analytically, according to the considered model, two rays arrive at the receiver antenna. We will denote the line of sight (LoS) ray electric field as $E_{\mathrm{LoS}}$ and the sea reflected ray field as $E_{\mathrm{ref}}$. The LoS ray will travel for $d_{\mathrm{LoS}}$ meters, which represents the distance between the two antennas, while the reflected ray will travel a longer distance $d_{\mathrm{ref}}$ that is easily calculable as

(4) $$d_{\mathrm{ref}}=\sqrt{4h^{2}+d_{\mathrm{LoS}}^{2}}.$$

Now, we will label the amplitude of the transmitted electric field with $E_{0}$, and we have a small angle Θ, due to the fact that the antennas’ height is small if compared with the antennas’ distance. This induces the neglect of the sea-parallel electric field component and allows us to write the total electric field that reaches the receiver antenna as

(5) $$\begin{array}{cc} E_{\mathrm{RX}} & =E_{\mathrm{LoS}}+E_{\mathrm{ref}}\\ \; & =E_{0}k_{\mathrm{LoS}}\cos\left(2\pi ft-\beta d_{\mathrm{LoS}}\right)+E_{0}k_{\mathrm{ref}}\cos\left(2\pi ft-\beta d_{\mathrm{ref}}\right), \end{array}$$

where $k_{\mathrm{LoS}}$ and $k_{\mathrm{ref}}$ are the attenuation operated by the radio medium to the LoS and reflected rays, respectively. A detailed discussion on the validity of the above expressions in the marine scenario we are considering here is reported in Appendix A.

It is worth saying that the two attenuation values $k_{\mathrm{LoS}}$ and $k_{\mathrm{ref}}$ depend on the distances $d_{\mathrm{LoS}}$ and $d_{\mathrm{ref}}$, respectively. Thus, we can express this dependence by writing $k_{\mathrm{LoS}}\to k_{\mathrm{LoS}}\left(d_{\mathrm{LoS}}\right)$ and $k_{\mathrm{ref}}\to k_{\mathrm{ref}}\left(d_{\mathrm{ref}}\right)$. Moreover, $\beta =\frac{2\pi }{\lambda }$, where $\lambda =\frac{c}{f}$ is the wavelength of the transmitted signal, *c* is the speed of light in the medium, and *f* is the signal frequency. Notice that $k_{\mathrm{ref}}$ contains the reflection coefficient operated by the air–sea separation surface. The channel medium effect can be calculated to normalize the received electric field with the transmitted amplitude $E_{0}$, which reads

(6) $$\varepsilon_{\mathrm{RX}}:=\frac{E_{RX}}{E_{0}}=k_{\mathrm{LoS}}\left(d_{\mathrm{LoS}}\right)\cos\left(2\pi ft-\beta d_{\mathrm{LoS}}\right)+k_{\mathrm{ref}}\left(d_{\mathrm{ref}}\right)\cos\left(2\pi ft-\beta d_{\mathrm{ref}}\right).$$

It is convenient to express the received electric field with the phasorial notation ${\hat{\varepsilon }}_{\mathrm{RX}}$, obtained via the well known Steinmetz transformation, which gives

(7) $${\hat{\varepsilon }}_{\mathrm{RX}}=k_{\mathrm{LoS}}\left(d_{\mathrm{LoS}}\right)e^{-i\beta d_{\mathrm{LoS}}}+k_{\mathrm{ref}}\left(d_{\mathrm{ref}}\right)e^{-i\beta \sqrt{4h^{2}+d_{\mathrm{LoS}}^{2}}}$$

where in the last equation we inserted the expression for $d_{\mathrm{ref}}$ calculated in (4).

The two-ray channel exhibits gain drops, which depend on the distance $d_{\mathrm{LoS}}$ as well as on the height *h* of the two antennas. The gain drops are amenable to the fact that the LoS ray and the reflected (and attenuated) one may arrive at the receiver antenna with a destructive phase difference of π radians. In order to qualitatively evaluate the channel behavior, it is of interest to calculate the magnitude of ${\hat{\varepsilon }}_{\mathrm{RX}}$. The channel effect results are

(8) $$\begin{array}{cc} \; & |{\hat{\varepsilon }}_{\mathrm{RX}}\left|=\right|k_{\mathrm{LoS}}\left(d_{\mathrm{LoS}}\right)e^{-i\beta d_{\mathrm{LoS}}}+k_{\mathrm{ref}}\left(d_{\mathrm{ref}}\right)e^{-i\beta \sqrt{4h^{2}+d_{\mathrm{LoS}}^{2}}}|\\ \; & =\sqrt{k_{\mathrm{LoS}}^{2}\left(d_{\mathrm{LoS}}\right)+k_{\mathrm{ref}}^{2}\left(d_{\mathrm{ref}}\right)+2k_{\mathrm{LoS}}\left(d_{\mathrm{LoS}}\right)k_{\mathrm{ref}}\left(d_{\mathrm{ref}}\right)\cos\left(\gamma (h,d_{\mathrm{LoS}},\lambda )\right)}, \end{array}$$

where

(9) $$\gamma \left(h,d_{\mathrm{LoS}},\lambda \right)=\beta \left(\sqrt{4h^{2}+d_{\mathrm{LoS}}^{2}}-d_{\mathrm{LoS}}\right).$$

It is easy to see that if $d_{\mathrm{LoS}}\gg h$, then the cosine argument tends to zero and $\cos\left(\gamma (h,d_{\mathrm{LoS}},\lambda )\right)≃1$, resulting in a channel response that is independent of the height of the antennas. Further, the channel would depend only on the expressions of the two parameters $k_{\mathrm{LoS}}\left(d_{\mathrm{LoS}}\right)$ and $k_{\mathrm{ref}}\left(d_{\mathrm{ref}}\right)$, which are functions of the antennas’ distance.

By imposing $\cos\left(\gamma (h,d_{\mathrm{LoS}},\lambda )\right)>0.9$, one derives

(10) $$\beta \left(\sqrt{4h^{2}+d_{\mathrm{LoS}}^{2}}-d_{\mathrm{LoS}}\right)<\frac{\pi }{7}$$

which, resolving the inequality for $d_{\mathrm{LoS}}$, gives

(11) $$d_{\mathrm{LoS}}>\frac{28h^{2}}{\lambda }-\frac{7\lambda }{14^{2}}≃\frac{28h^{2}}{\lambda }.$$

We can assume we are in the far-field region when $d_{\mathrm{LoS}}>10\lambda$, which, according to Equation (11), implies

(12) $$h>\sqrt{\frac{5}{14}}\lambda ≃0.6\lambda .$$

Equations (11) and (12) provide rule-of-thumbs for the possible geometry of the system.

### 3.1. Large-Scale Channel Model and Path Loss Exponent

The propagation of a radiated signal from a transmitter antenna in the void can be expressed as the power of the electromagnetic wave calculated at the distance *d* and can be modeled with the well known Friis formula

(13) $$P_{\mathrm{RX}}\left(d\right)=P_{\mathrm{TX}}G_{\mathrm{TX}}G_{\mathrm{RX}}{\left(\frac{\lambda }{4\pi d}\right)}^{2},$$

where $P_{\mathrm{TX}}$ is the total transmitted power, and $G_{\mathrm{TX}}$ and $G_{\mathrm{RX}}$ are the transmitter and receiver antenna gains in the direction of the segment connecting the two antennas, respectively. In this case, if the Expression (13) is expressed in logarithmic units (dB), it is clearly seen that the received power decays, as a function of *d*, with a slope of 20 dB/decade. In a more realistic environment, where the channel is a propagation medium with losses, the received power can decay faster than 20 dB for each distance decade. Hence, let us consider the large-scale model for the received power $P_{\mathrm{RX}}$ presented in [7]

(14) $$P_{\mathrm{RX}}\left(d\right)=P_{\mathrm{RX}}\left(d_{0}\right){\left(\frac{d_{0}}{d}\right)}^{n}.$$

The parameter $d_{0}$ is a reference distance in the far-field radiation region. As outlined above, we can use Equation (11) and assume a reference distance calculated as

(15) $$d_{0}\geq \frac{28h^{2}}{\lambda },$$

as expressed in (9). By defining the path loss (PL) at distance *d* as

(16) $$\mathrm{PL}\left(d\right)=\frac{P_{\mathrm{TX}}}{P_{\mathrm{RX}}\left(d\right)},$$

and inserting (14) into (16), we obtain an expression for the PL that is relative to the reference distance $d_{0}$:

(17) $$\mathrm{PL}\left(d\right)=\mathrm{PL}\left(d_{0}\right){\left(\frac{d}{d_{0}}\right)}^{n}.$$

The PL exponent plays a key role for the large-scale channel characterization, and our goal is to retrieve it via an on-site measurements campaign. Equation (17) can be expressed in decibels for better reading, obtaining [15]

(18) $$\begin{array}{cc} {\mathrm{PL}}^{\left(dB\right)}\left(d\right) & ={\mathrm{PL}}^{\left(\mathrm{dB}\right)}\left(d_{0}\right)+10\;n\log_{10}\left(\frac{d}{d_{0}}\right)\\ \; & =k_{1}+10\;n\log_{10}\left(d\right). \end{array}$$

Since the term $k_{1}={\mathrm{PL}}^{\left(\mathrm{dB}\right)}\left(d_{0}\right)-10\;n\log_{10}\left(d_{0}\right)$ does not depend on the distance *d*, the PL exponent actually represents the slope of ${\mathrm{PL}}^{\left(\mathrm{dB}\right)}\left(d\right)$ in (18). Another key point for the large-scale propagation behavior to be taken into consideration is the random nature of the path loss measurements. In fact, in accordance with [7], in a real scenario, we can see a random dispersion of the PL values when the antennas’ distance is maintained fixed. For this reason, the PL expression should include a random variable that models the power oscillations around the PL mean value. Let us call ${\mathrm{PL}}_{M}\left(d\right)$ an instance of a set of path loss measures (in dB) in the considered environment. Considering (18), it is possible to model the actual measurements as

(19) $${\mathrm{PL}}_{M}\left(d\right)={\mathrm{PL}}^{\left(\mathrm{dB}\right)}\left(d\right)+\rho_{M},$$

where $\rho_{M}$ is a zero-mean Gaussian random variable [7] with variance $\sigma_{\rho }^{2}$. Basically, ${\mathrm{PL}}_{M}\left(d\right)$ is a Gaussian random variable due to the presence of $\rho_{M}$, with its mean represented by ${\mathrm{PL}}^{\left(\mathrm{dB}\right)}\left(d\right)$. The validation of the Gaussian nature of the random variable $\rho_{M}$ is given in the Section 5 of this paper, where we focus on the statistical properties of the collected data. The PL exponent *n* and the parameter $k_{1}$ of (18) can be retrieved with a minimum mean square error fit of the measured data. Furthermore, it is easy to retrieve the variance $\sigma_{\rho }^{2}$ of $\rho_{M}$ with a minimum mean square error fit for the zero-mean random values ${\mathrm{PL}}_{M}\left(d\right)-{\mathrm{PL}}^{\left(\mathrm{dB}\right)}\left(d\right)$.

## 4. Path Loss Measurement Campaign Results

**Figure 9:**
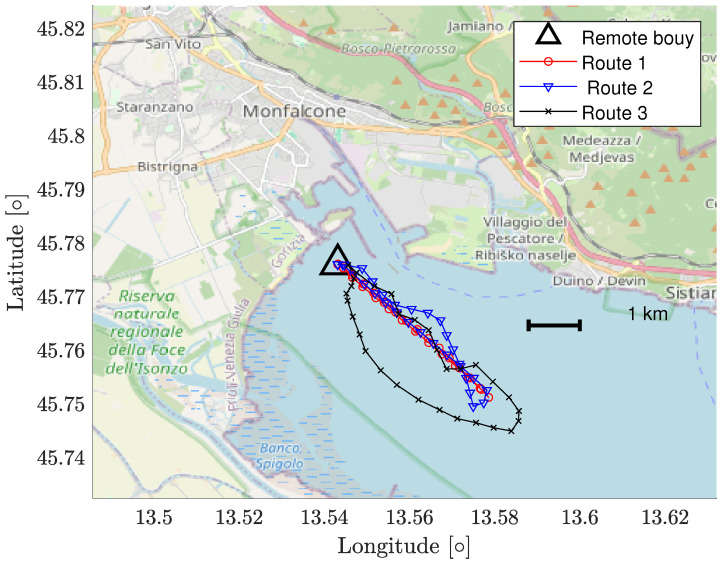
Routes followed in the measurement campaign. The scale is also expressed.

In order to carry out an analysis on the electromagnetic propagation in the considered marine environment, we equipped the sail boat test bed with a precision GPS receiver, which returns the latitude and longitude of the sail boat on a USB/COM port every second. The received power is logged for each GPS point. The received signal strength intensity (RSSI) is contained in each received packet sent from the radio modules to the Arduino board. The RSSI and GPS data collection starts from the buoy’s location; thus, the remote communication node’ position is retrieved as the first logged GPS information. We selected three routes to be followed, which are depicted in Figure 9.

**Figure 10:**
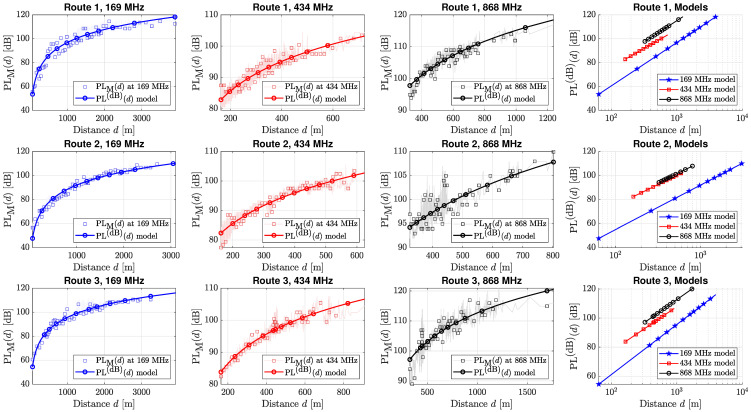
Measured and modeled PL at the three different ISM sub-gigahertz bands. The rows refer to the different routes, while the columns refer to the considered ISM bands, as reported in each figure’s title. The fourth column aggregates the evaluated PL models at the three different ISM bands for comparison.

The dates in which the measurement campaigns have taken place are 14 October, 18 October, and 23 November 2022, for Route 1, Route 2, and Route 3, respectively. The position of the remote buoy is the same for each route. The results in terms of measured PL retrieved from RSSI log files are depicted in Figure 10, where we started to consider RSSI values to be valid for distances that are greater than the threshold expressed in (11).

The plots in Figure 10 show that not all the RF modules are suitable to reach long distances. In fact, despite the differences in the PL due to the transmitted frequency, the data collected with the 169 MHz ISM band covers a distance up to 4 km, while the 434 MHz radio link can reach up to 800 m. This behavior is amenable to the different sensitivities that characterize the deployed RMs. As also expected from (13), the lower the frequency, the lower the PL is, when a fixed distance from the communication nodes is considered. In particular, the 169 MHz ISM band sees a decrement of 12 to 19 dB with respect to the PL experimented at the 434 MHz ISM band, and a decrement of 17 to 20 dB with respect to the PL experimented at the 868 MHz ISM band.

## 5. Statistical Analysis of the Collected Data

In this section, we would like to focus on the statistical nature of the collected data used for the PL calculation. The first important parameter to be investigated is the average value of the evaluated PL exponent. Indeed, this parameter could give a precise idea of the issues that a sub-gigahertz radio communication could encounter in the marine environment. Table 3 shows the computed PL exponents for the different routes at the three different ISM bands, and the average value $\bar{n}$ for each route.

The values are strictly correlated to the results obtained in [16], where the overall average PL exponent in indoor environments is 3.14. In particular, we can state that the PL exponent in the marine environment is similar to the one experimented on at 914 MHz with the two communication nodes placed on the same floor of an offices building. Conversely, in [17], the authors obtained PL exponent values that are in the range of 2.21–2.25 for the 850 MHz 5G band, in a terrain-based outdoor environment. This suggests that the marine scenario introduces higher propagation losses if compared with a terrestrial propagation environment. The average value for the PL exponent is not sufficient to completely model the power losses in the considered propagation environment. In fact, the variance of the PL fluctuations also takes part in the statistical characterization of the path loss. In a first attempt to do so, we can aggregate all the data collected for each route and each frequency, and we estimate the variance $\sigma_{\rho }^{2}$ (shown in Table 4) of the collected PL measurements, according to Model (19).

At a first glance, the obtained variances exhibit great differences in their values. For example, the $\sigma_{\rho }^{2}$ for Route 1 at the 169 MHz ISM band is much greater than the other values at the same ISM band.

**Figure 11:**
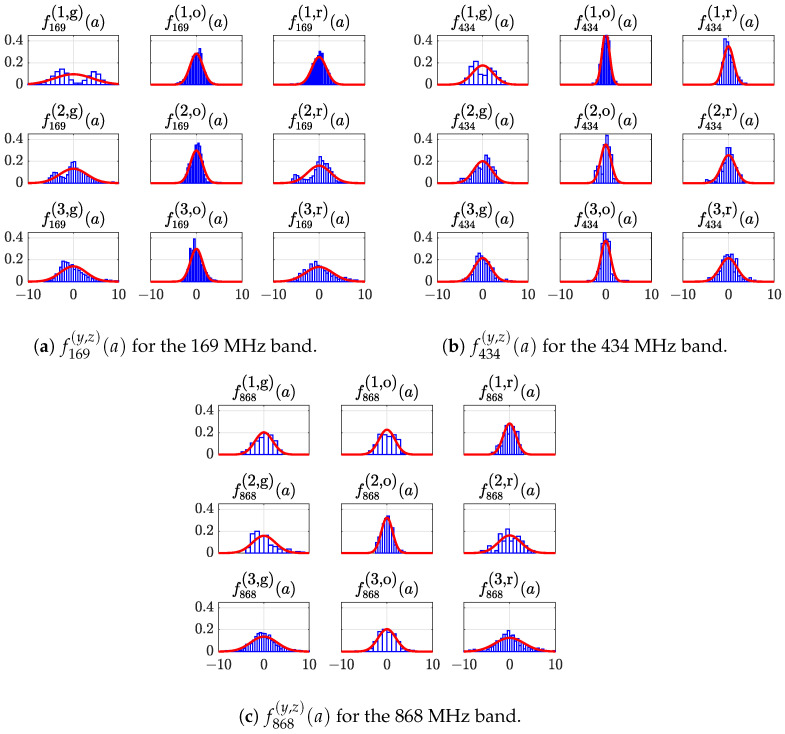
Empirical (bluish bars) and fitting (red solid line) statistical distribution histograms of the PL fluctuations for the 169 MHz (**a**), 434 MHz (**b**), and 868 MHz (**c**) ISM band measurements. The data are also subdivided in PL fluctuations that refer to the aggregated, outward route and return route, respectively.

A deeper glance at the data statistical distribution for the fluctuations $\rho_{M}$ carried out for the measurements for each Route and ISM band shows the actual PL fluctuations’ statistical nature. In fact, the results, in terms of probability density function $f_{x}^{\left(y,z\right)}\left(a\right)$, are depicted in Figure 11a–c, where the subscript *x* indicates the considered band, i.e., the x ∈ { 169, 434, 868 } MHz band; the first superscript *y* indicates the route, i.e., y ∈ { 1,2,3 }, and the second superscript *z* indicates if the data refer to the aggregated data (g), the outward route data (o), and the return route data (r).

**Figure 12:**
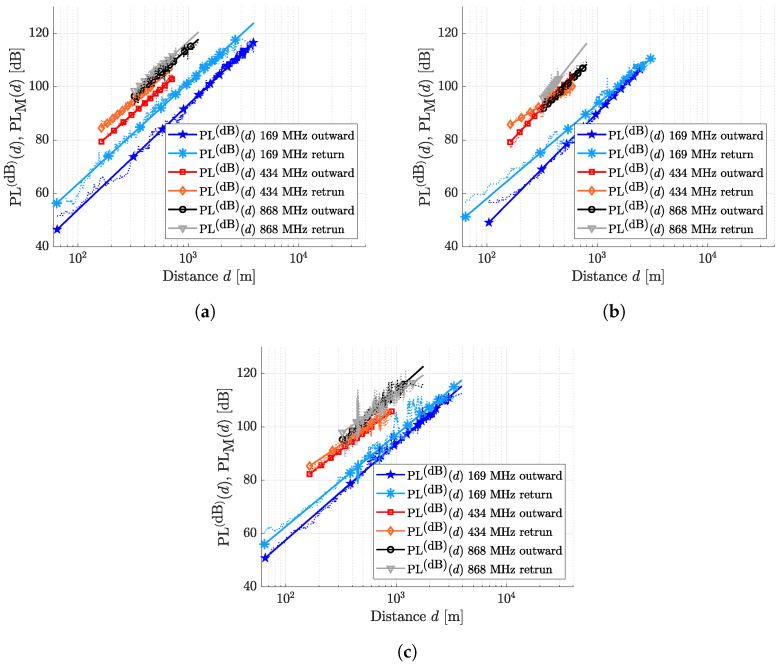
Measured and modeled PLs distinguished for the three different ISM bands, referring to the outward and return paths followed for Route 1 (**a**), Route 2 (**b**), and Route 3 (**c**).

Figure 11a–c show the actual statistical distributions histograms (bluish bars) and the Gaussian fitting distribution (red solid line) for the PL fluctuations at 169 MHz, 434 MHz and 868 MHz, respectively. More precisely, the second column of each of the figures refers to the PL fluctuations’ distribution, which refers to the outward part of each route (the path followed by the boat in the outward direction, from the remote buoy towards the sea horizon). The third column refers to the PL fluctuations’ distribution of the return part of each route (the path followed by the boat to return at the remote buoy’s position). The first column of the figure is the aggregated PL fluctuations, i.e., the union of the outward and return route measured PL fluctuations. It is worth noting that the aggregated distributions of the measured PL fluctuations can exhibit histograms that are not fitted by the Gaussian function, while the separated outward and return routes are correctly fitted. This behavior suggests that the propagation mechanisms are different for the outward and return routes, respectively. In order to have a complete understanding of the electromagnetic propagation over the considered routes, it is helpful to look at Figure 12a–c.

Route 1, for example, exhibits two distinct received power curves at the 169 MHz ISM band: upon initial inspection, the outward route displays higher received power compared to the return route. This is attributed to the fact that the paths are perfectly radial with respect to the remote buoy, as depicted in Figure 9 of the paper. As the on-boat antennas are positioned at the back side of the boat, the received power for the outward route is higher because the on-boat antenna directly receives signals from the remote buoy’s antenna without any obstacles in between. Conversely, when considering the return route, the metallic structures (such as the ship’s rail and boat mast) of the boat are situated exactly between the two radio nodes. As an initial approximation, due to the large wavelength of the signals in the 169 MHz band, the metallic structures function as a shield for the electromagnetic field. With higher frequencies, the wavelength decreases, and the electric dimensions of the metallic structures increase. Consequently, as the frequency increases, the propagation mechanisms induced by the boat’s metallic structures transition from shielding to scattering.

For this reason, the aggregate dispersion of the PL fluctuations around the average PL curve exhibits “double statistical behavior”, more noticeable at lower frequencies, when the antennas are blinded by the metallic obstacles. In particular, the higher the shielding (i.e., the metallic structures’ density that blind the two antennas), the more evident is the double peak shape for the measured PL fluctuations histogram. The confirmation comes by considering the histogram for Route 3, 169 MHz aggregate (Figure 12a). Differently from the sharp path of Route 1, in this case the antennas are not fully blinded by the boat at the return path. Consequently, the PL fluctuations show a smooth statistical dispersion, tending to the expected Gaussian-like distribution. In the Table 5, we finally give the variance ${\left(\sigma_{\rho ,x}^{\left(y,z\right)}\right)}^{2}$ of the probability density functions $f_{x}^{\left(y,z\right)}$ for all the outward and return routes depicted in Figure 11a–c.

## 6. Impact of Weather Conditions

**Figure 13:**
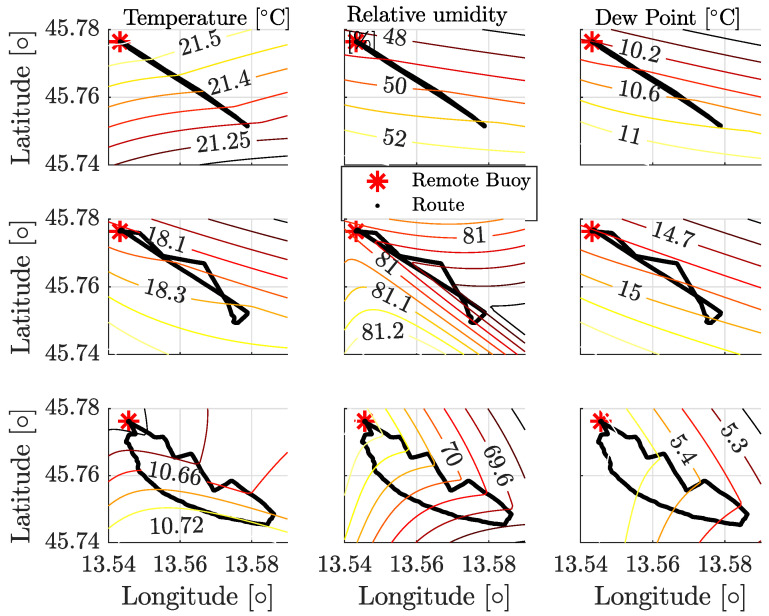
Weather conditions (temperature, relative humidity, and dew point) interpolated from the data collected by the weather stations in the surroundings of the routes depicted in Figure 9.

**Figure 14:**
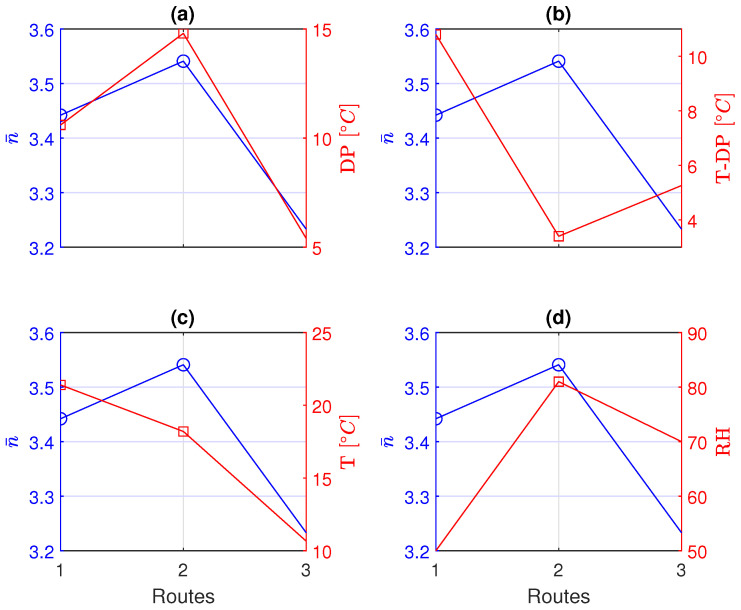
Averaged PL exponent compared with the weather parameters: (**a**) dew point; (**b**) excess temperature from the dew point; (**c**) temperature; and (**d**) relative humidity.

The averages $\bar{n}$ of the PL exponents presented in Table 2 show great variability. As a possible cause, we considered the weather conditions present when the measurements have been performed. Hence, we are interested in correlating the PL exponents with the weather conditions stored by four regional weather stations [18], placed in the surroundings of the routes. The coordinates of the four weather stations are [45.618291 N, 13.565022 E], [45.649996 N, 13.752242 E], [45.714768 N, 13.458865 E], and [45.780471 N, 13.536554 E]. To characterize the weather conditions, we focused on the temperature (T), relative humidity (RH), and dew point (DP) [19], which are interpolated and plotted over the routes of Figure 9 as shown in Figure 13. The averaged PL exponent $\bar{n}$, evaluated over the three different ISM bands for each route, is now considered and correlated with the weather parameters. In particular, we correlate the average PL exponent $\bar{n}$ with the dew point (Figure 14a), with the difference between the temperature and the dew point (excess temperature, Figure 14b), with the temperature (Figure 14c), and with the relative humidity (Figure 14d), respectively.

We observe that the experimental results show a strong correlation between the averaged values of the PL exponent with the dew point. In particular, the higher the dew point, the higher the received power decay slope.

## 7. Conclusions

This paper focused on the design of a test bed in order to perform a path loss (PL) measurement campaign in three different sub-gigahertz industrial, scientific, and medical (ISM) bands for low power near-sea surface communications. The results indicate that the PL exponents are greater than three in each ISM band, for each of the three routes we considered. In accordance with the Friis formula for the radio link, we observed that an intrinsic gain in the propagation can be obtained by lowering the transmission frequency. In fact, the PL for the 169 MHz band is 15 dB lower than the one measured in the other two considered bands. Moreover, we also highlighted the fact that the propagation is affected by the weather conditions since we observed that the experimental results show a clear correlation between the PL exponent and the dew point. In particular, the higher the dew point, the higher is the received power decay slope.

## Figures and Tables

**Table 1:** Dipole system gain obtained via simulations, for the three different ISM bands.

Parameter	169 MHz	434 MHz	868 MHz
	ISM Band	ISM Band	ISM Band
$G_{avg}$	2.32 dB	2 dB	2.03 dB
$G_{min}$	2.27 dB	1.42 dB	0.66 dB
$G_{max}$	2.37 dB	2.75 dB	3.43 dB

**Table 2:** OBW and ChP measurement parameters and results.

Parameter	169 MHz	434 MHz	868 MHz
	ISM Band	ISM Band	ISM Band
Res. bandwidth	300 Hz	1 kHz	1 kHz
Video bandwidth	1 kHz	3 kHz	3 kHz
Frequency span	600 kHz	600 kHz	600 kHz
OBW	14.5 kHz	78 kHz	78 kHz
ChP	14.1 dBm	13.7 dBm	13.8 dBm

**Table 3:** Best-fitting path loss exponents *n* for the three considered bands, computed for the three routes of Figure 9.

Route	*n* at 169 MHz	*n* at 434 MHz	*n* at 868 MHz	$\bar{n}$
	ISM Band	ISM Band	ISM Band	
1	3.629	3.146	3.551	3.442
2	3.699	3.695	3.455	3.541
3	3.447	3.223	3.189	3.233

**Table 4:** $\rho_{M}$ variance calculated for each ISM band and each route.

Route	$\sigma_{\rho }^{2}$ at 169 MHz	$\sigma_{\rho }^{2}$ at 434 MHz	$\sigma_{\rho }^{2}$ at 868 MHz
	ISM Band	ISM Band	ISM Band
1	17.55	5.171	3.845
2	9.157	3.956	6.365
3	8.111	3.400	8.543

**Table 5:** $\rho_{M}$ variance calculated for each ISM band and each outward and return route.

Route	${\left(\sigma_{\rho ,169}^{\left(y,z\right)}\right)}^{2}$	${\left(\sigma_{\rho ,434}^{\left(y,z\right)}\right)}^{2}$	${\left(\sigma_{\rho ,868}^{\left(y,z\right)}\right)}^{2}$
	ISM Band	ISM Band	ISM Band
1 outward	1.97	0.67	3.07
1 return	2.49	1.29	1.97
2 outward	1.78	1.27	1.51
2 return	6.26	2.43	6.12
3 outward	1.76	1.14	3.79
3 return	8.83	3.48	10.13

**Figure A1:**
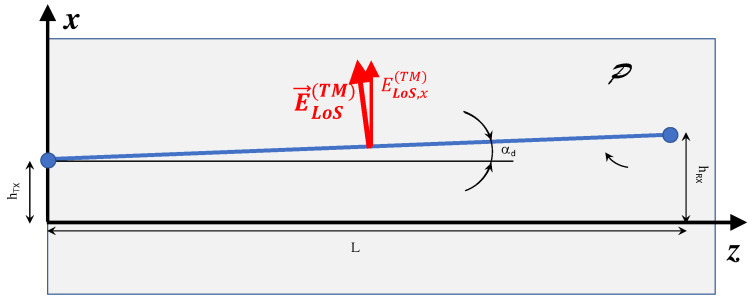
Schematic diagram of waves propagating from the transmitter to the receiver along the line of sight (LoS) direction.

**Figure A2:**
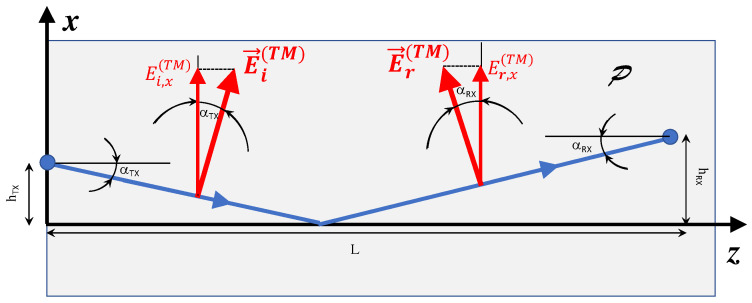
Schematic diagram of waves propagating from the transmitter to the receiver through reflection at the air–sea interface.

**Figure A3:**
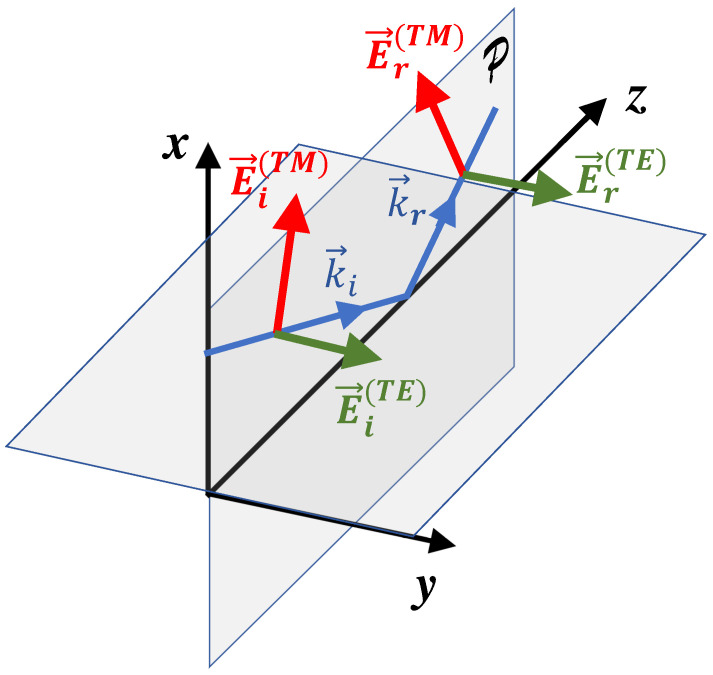
Schematic diagram for the separated TE and TM contributions of waves propagating from the transmitter to the receiver through reflection at the air–sea interface.

## Data Availability

Data are contained within the article.

## Abbreviations

ChP	Channel power
DP	Dew point
IL	Insertion loss
IoT	Internet of Things
ISM	Industrial, scientific, and medical bands
LAN	Local area network
LoS	Line of sight
OBW	Occupied bandwidth
PL	Path loss
PSD	Power spectral density
RF	Radio frequency
RH	Relative humidity
RL	Return loss
RM	Radio module
RSSI	Received signal strength intensity
SMA	SubMiniature version A connector
T	Temperature
TE	Transverse electric
TEM	Transverse electro-magnetic
TM	Transverse magnetic
UART	Universal asynchronous receiver-transmitter
VNA	Vector network analyzer

## Mathematical Symbols

*n*	Path loss exponent
$\rho_{M}$	Zero-mean Gaussian random variable for modeling the path loss fluctuations
$f_{x}^{\left(y,z\right)}$	Probability density function for the *x* ISM band, referring to the *y*-th outward (z=o)
	or return (z=r) route
$G_{TX},G_{RX}$	Transmitter and receiver antenna gain
$\sigma_{\rho }^{2}$	Variance of the path loss fluctuations, i.e., $\rho_{M}$ Gaussian random variable
${\left(\sigma_{\rho ,x}^{\left(y,z\right)}\right)}^{2}$	Variance of the path loss fluctuations for the *x* ISM band, referring to the *y*-th outward
	(z=o) or return (z=r) route
${\mathrm{PL}}_{M}\left(d\right)$	Modeled path loss expressed in dB, as a function of the antennas’ distance
$P_{RX}\left(d\right)$	Received power at the distance *d*
$Z_{0}$	Reference impedance for the IL and RL parameters

## Appendix A. A Focus On The Proposed Propagation Model

Recall that in a radio link the amount of power received from the receiving antenna is

(A1) $$P_{RX}\propto {\vec{E}}_{inc}\cdot {\vec{\ell }}_{RX}$$

where ${\vec{E}}_{inc}$ is the (vector) electric field impinging on the receiving antenna and ${\vec{\ell }}_{RX}$ is the receiving antenna effective length (see, for instance, [9], Chapter 2.15). Let us begin our analysis from an ideal case where:

- The sea is totally flat and is regarded as a perfect electric reflector;
- The antenna on the transmitting boat is perpendicular to the sea;
- Also, the antenna on the receiving buoy is perpendicular to the sea.

The figures below depict such an ideal scenario, illustrating both the direct line-of-sight (LoS) wave reaching the receiver (Figure A1) and the wave after reflection on the sea surface (Figure A2).

For enhanced clarity, Figure A3 presents the three-dimensional representation of the reflected wave.

We denote the plane containing the incident and reflected wave vectors (${\vec{k}}_{i}$ and ${\vec{k}}_{r}$, respectively) as P and refer to the waves whose electric field lies within plane P or is orthogonal to it as transverse magnetic (TM) and transverse electric (TE), respectively.

In the ideal scenario under examination, the field is entirely TM-polarized because the transmitting antenna generates a vertically polarized field, and the reflection from the flat sea does not introduce any TE-polarized component. Strictly speaking, neither the direct ray nor those incident (on the sea surface) and reflected (from the sea surface) are exactly vertical. The rigorous evaluation of the received power would hence require the computation of the inner product ${\vec{E}}_{inc}\cdot {\vec{\ell }}_{RX}$ as in Equation (A1) above. However, in the scenario addressed in this article, regarding ${\vec{E}}_{inc}$ and ${\vec{\ell }}_{RX}$ as vertical vectors, assessing the received power through a scalar approach as in Equation (5) of the main text introduces an error of completely negligible magnitude. In fact, the heights of the transmitting antennas ($h_{TX}$ and $h_{RX}$ in the figures) are approximately 1–2 m, while the distance between the transmitter and receiver (L in the figure) is on the order of 100–1000 m. Consequently, the angles $\alpha_{TX}$, $\alpha_{RX}$ and $\alpha_{LoS}$ are in the order of tenths of a degree. Thus, both the field ${\vec{E}}_{inc}$ impinging on the receiving antenna and the antenna effective length ${\vec{\ell }}_{RX}$ can be regarded as vertical vectors within an error that approximates $1-\cos\left(\alpha \right)\approx \alpha^{2}/2$, i.e., roughly on the order of 1 part in 10,000.

We now extend the study from the ideal case to a more realistic scenario in which, due to the wave motion of the sea, the transmission and reception antennas may oscillate, no longer remaining precisely vertical relative to the ground. Additionally, the reflection from the sea surface, if not flat, may introduce components with TE polarization. This would cause the field that reaches the receiver to be elliptically polarized.

The cumulative effect of these oscillations results in a decrease in the signal received by the receiving antenna because the vectors ${\vec{E}}_{inc}$ and ${\vec{\ell }}_{RX}$ are no longer precisely aligned in parallel. In an equivalent manner, the decrease in received signal can be attributed to an increase in path-loss between the transmitting and receiving antennas. In this sense, the simple scalar expressions given in Equation (5) can also account for fluctuations in the orientation of the antennas and thus for the loss of received power due to the appearance of the TE component and the imperfect alignment between ${\vec{E}}_{inc}$ and ${\vec{\ell }}_{RX}$. In fact, the fluctuations result in a random increase in propagation losses, which can be incorporated into the general approach presented in Section 3.1 of the main text.
